# Insights Into Host Cell Cytokines in *Chlamydia* Infection

**DOI:** 10.3389/fimmu.2021.639834

**Published:** 2021-05-21

**Authors:** Wenjing Xiang, Nanyan Yu, Aihua Lei, Xiaofang Li, Shui Tan, Lijun Huang, Zhou Zhou

**Affiliations:** ^1^ Institute of Pathogenic Biology, Hengyang Medical College, Hunan Provincial Key Laboratory for Special Pathogens Prevention and Control, Hunan Province Cooperative Innovation Center for Molecular Target New Drug Study, University of South China, Hengyang, China; ^2^ Nanyue Biopharmaceutical Co. Ltd., Hunan Province Innovative Training Base for Postgraduates, University of South China and Nanyue Biopharmaceutical Co. Ltd., Hengyang, China

**Keywords:** *Chlamydia*, infection, cytokines, inflammation, pathology

## Abstract

Chlamydial infection causes a number of clinically relevant diseases and induces significant morbidity in humans. Immune and inflammatory responses contribute to both the clearance of *Chlamydia* infection and pathology in host tissues. *Chlamydia* infection stimulates host cells to produce a large number of cytokines that trigger and regulate host immune responses against *Chlamydia*. However, inappropriate responses can occur with excessive production of cytokines, resulting in overreactive inflammatory responses and alterations in host or *Chlamydia* metabolism. As a result, *Chlamydia* persists and causes wound healing delays, leading to more severe tissue damage and triggering long-lasting fibrotic sequelae. Here, we summarize the roles of cytokines in *Chlamydia* infection and pathogenesis, thus advancing our understanding chlamydial infection biology and the pathogenic mechanisms involved.

## Introduction

*Chlamydia* are gram-negative prokaryotic organisms with obligate intracellular parasitism (1). Each species of *Chlamydia* is capable of infecting the host species—ranging from humans to amoebae—that they are adapted to (2). In animals, infections with *Chlamydia* can result in inflammatory pathologies at the sites of infection, including ocular, pulmonary, genital, articular, and intestinal tissues. *Chlamydia* infections and their transmission impose a significant medical and social burden, thus causing economic damage and representing a major public health challenge (3), and there is currently no optimal strategy to control chlamydial infections and stop their spread. Although chlamydial vaccine research dates to seventy years ago, an effective vaccine is not yet available for the limitations in the safety and protective immunity (4). Drug therapy is beneficial for temporary control of infection but unable to treat the irreversible lesions caused by reinfection and persistent asymptomatic infection (5). Therefore, it is crucial to deeply investigate the pathogenic mechanisms of *Chlamydia* to develop more effective strategies for the treatment and prevention of these diseases.

*Chlamydia* have a biphasic life cycle, alternating between the infectious elementary body (EB) and the replicative reticulate body (RB). Intracellular infection starts with the entry of EBs into a host cell. Then, the endocytosed EBs differentiates into noninfectious but metabolically active RBs (6), which replicates and converts into EBs again for transmission of the infection to a new host cell (1). Invasion of the host by *Chlamydia* and the ensuing chlamydial life cycle, involves series of poorly understood mechanisms that compromise and interfere with the function of the host cells, thus damaging host health. Instead, it is critical for the host to mount an immune response, including production of cytokines such as interleukin (IL)-1, IL-6, IL-8, and tumor necrosis factor alpha (TNF-α) that activate or recruit immune cells to trigger or amplify inflammation against *Chlamydia* (7, 8). These cytokines can be not only used by immune system to inhibit *Chlamydia* growth and control infection, which is helpful for preventing or slowing down the progression of chlamydial lesions (9, 10), but also used for microbial survival but not for clearance, and result in irreversible lesions and severe tissue damage (**Table 1**).

**Table 1:** Function of cytokines in pathological changes during *Chlamydia* infection.

Cytokine	Methods for research	Regulatory role in host immune response	Function in pathology
IL-1	Chemical inhibition, antibody blockade and KO mice (11, 12)	Regulate Th1/Th2 balance Regulate other pro-inflammation cytokines	Accelerate formation of tissue lesions
IL-6	Chemical inhibition, antibody blockade and KO mice (13, 14)	Recruit white cell Promote B cell differentiation Regulate Th1 response	Induced pathology has been controversial
IL-8	KO mice (15)	Inhibit apoptosis of neutrophils Reduce sensitivity of therapeutic drugs	Accelerate formation of tissue lesions
IL-13	KO mice (16)	Regulate other pro-inflammation cytokines	Accelerate tissue lesion
IL-17	Antibody blockade and KO mice (17)	Up-regulate iNOS and NO Regulate DC to evoke Th1 response Increase neutrophil infiltration	Accelerate formation of tissue lesions
IL-4	Antibody blockade and KO mice (18)	Enhance B cell to present antigen Trigger DTH Lessen inflammatory	Accelerate formation of tissue lesions
IL-10	siRNA inhibition, chemical inhibition, antibody blockade and KO mice (19)	Down-regulated the expression of MHC I molecules Inhibit apoptosis of DC and control its antigen-presentation function Reduced local inflammatory infiltration Decreased eliminating activity of CD8^+^ T cells	Attenuate pathological damage
INF-γ	siRNA inhibition, chemical inhibition, antibody blockade and KO mice (20–22)	Inhibit host cell metabolism Regulate Th1/Th2 balance Influence *Chlamydia* life cycle	Clear infection and reduce sequelae
TNF-α	siRNA inhibition, chemical inhibition, antibody blockade and KO mice (23–25)	Inhibit host metabolism Induce apoptosis of infected cells Enhance neutrophil and macrophage phagocytic activity Facilitate the expression of other cytokines	Involved in immune injury

**Figure 1:**
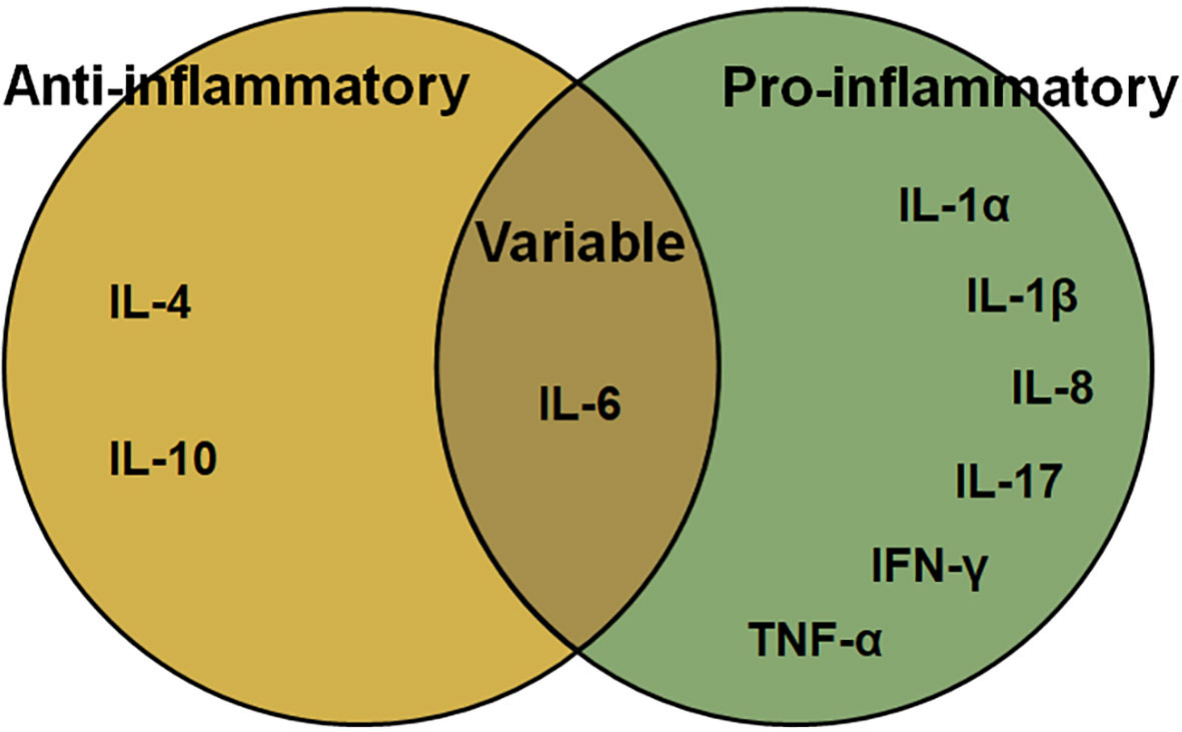
The function of cytokines in *Chlamydia*-induced inflammation.

*In vitro* and *in vivo* studies on *Chlamydia* infection show that a variety of cytokines, including IL, interferon (IFN), and TNF are involved in the inflammatory response (**Figure 1**) and immune regulation in *Chlamydia*-induced diseases. Here, we attempt to summarize the roles of cytokines involved in *Chlamydia* infection and pathogenesis.

## IFN

IFN was originally shown to induce an antiviral state in host cells, and was later found to be a cytokine with several effects on the immune system (26, 27). Type I interferon (IFN-α/β) mainly functions as an antiviral factor and immune regulator (26, 28). Type II interferon (IFN-γ) is involved in immunoregulation, and has anti-bacterial, anti-parasitic and anti-tumor functions (26, 27).

The level of IFN is elevated in the cell culture supernatants, serum, and cervical secretions after *Chlamydia* infection (20, 29, 30). During chlamydial infection, IFN-α/β activates macrophages, enhances the cytotoxic activity of natural killer (NK) cells, and promotes IFN-γ production or Th1 cell differentiation through the activator of transcription (STAT) signal pathway (31). However, the precise role of IFN-α/β in chlamydial infection is not very clear (32). IFN-γ plays an anti-*Chlamydia* role in the innate immune system and adaptive immune system. The secretion of IFN-γ is not only regulated by IL-12, IL-18, IL-10, and other cytokines after chlamydial infection, but is also enhanced through a positive feedback mechanism (33–35). The importance of IFN-γ in the host during chlamydial infection is evidenced by the elevated chlamydial load in IFN-γ ^-/-^, IFN-γR ^-/-^ mice or mice treated with anti-IFN-γ antibody compared with that in the wild/control group (20–22).

IFN-γ inhibits the normal metabolism and replication of *Chlamydia* by affecting availability of essential nutrients for *Chlamydia* growth. IFN-γ not only strongly reduces *C. trachomatis* metabolic growth *via* cellular tryptophan depletion and glucose starvation (36), but also interferes with the iron metabolism of the host (37). In addition, IFN-γ has immune-defensive functions in the host. Severe combined immunodeficiency (SCID) mice treated with neutralized anti-IFN-γ antibody, or RAG-1^-/-^/IFN-γR^-/-^ mice exhibit increased susceptibility to *C. trachomatis* compared with RAG-1^-/-^ mice, suggesting that IFN-γ exerts beneficial effects on host innate immunity for controlling *Chlamydia* infection (38). Furthermore, the role of IFN-γ against *Chlamydia* in adaptive immune protection can be demonstrated by transfer of *Chlamydia*-specific CD4^+^ or CD8^+^ T cells, which induce IFN-γ production after infection and provide complementary mechanisms for maintaining protective levels of IFN-γ (39). *Chlamydia*-specific CD8^+^ T cells, derived from IFN-γ-deficient mice, failed to provide protection under conditions whereas the wild-type CD8^+^ T cells did (40, 41). IFN-γ also has a regulatory impact on anti-*Chlamydia* defense by altering the Th1/Th2 balance, which is modulated by STAT1 phosphorylation and subsequent activation of the Th1/Th2 cell differentiation-specific transcription factor T-bet (42, 43). However, low-level IFN-γ induces the formation of smaller atypical inclusions that contain large RBs and non-replicating aberrant bodies with no newly generated EBs, which are associated with the persistent infection of *Chlamydia* (32, 44).

IFN-γ not only has an anti-*Chlamydia* function, but also influences the outcome of *Chlamydia* infection. Under normal conditions, IFN-γ can accelerate the clearance of *Chlamydia*, control infection effectively, and alleviate the lesions that form. However, a high concentration of IFN-γ is related to excessive inflammatory reaction and infectious sequelae (45, 46). Thus, the diverse effects of IFN-γ on *Chlamydia* infection-induced immune response are related to its concentration, the immune microenvironment, and the stage of infection (32, 33). Anti-*Chlamydia* strategies seek to take advantage of the functions of IFN-γ: for example, cell-specific IFN-γ/IFN-γR gene knockout (KO) mice may be established using the Cre/loxP recombinant system, defining where IFN-γ exerts its anti-infective effects. Furthermore, magnifying the effects of cell-targeting IFN-γ in that particular area can be used to improve the sensitivity of IFN-γ treatment. Genital and intestinal epithelial cells should be the main focus in strategies against *C. trachomatis* infection.

## TNF

TNF, an important inflammatory factor mainly produced by activated monocytes/macrophages (47), is divided into the categories of TNF-α and TNF-β (48). TNF-α is chiefly secreted in response to inflammatory stimuli and is well known for its killing effect against intracellular microbes (23). *Chlamydia* or its pathogenic substances such as pORF5 and lipopolysaccharide (LPS) induce TNF-α production in host serum, bronchial lavage fluid, vaginal secretions, and supernatants of cultured cells cells (49–51), through activating mitogen-activated protein kinases (MAPK) or other signaling pathways (52, 53). Toll-like receptor (TLRs) regulate TNF-α expression. Deficiency of TLR2 or TLR4 in macrophages significantly reduces TNF-α levels during chlamydial infection (53, 54),while deficiency of TLR3 in epithelial cells increases its levels at the early stage of chlamydial infection (55).

Elevated TNF-α levels have certain effects on *Chlamydia* infection. First, TNF-α works with IFN-γ to inhibits the metabolism of host cells by increasing the activity of indoleamine 2, 3-dioxygenase (IDO), and restricts the growth of *Chlamydia* (36, 56). Secondly, TNF-α influences the survival of *Chlamydia in vivo* by inducing apoptosis of host cells that provide suitable conditions for the growth of the organism (57–59). Furthermore, the adoptive transfer of TNF-α reduces the lung bacterial load of newborn mice infected with *Chlamydia*. It is possible that TNF-α strongly enhances the phagocytic activity of neutrophils and macrophages (60). However, deficiency of TNF-α or application of TNF-α inhibitors has no significant effect on *Chlamydia* clearance *in vivo*, suggesting that TNF-α may not be necessary for *Chlamydia* clearance (23).

It has been documented that TNF-α is related to the formation of *Chlamydia*-induced lesions. TNF-α not only up-regulates adhesion molecules, assisting the exudation of leukocytes to the site of inflammation, but also facilitates the expression of cytokines such as IL-6 and IL-8, which are connected with tissue fibrosis and scar formation (61, 62). Second, TNF-α accelerates the release of collagenase by stimulating the proliferation of tissue fibroblasts, which leads to histopathological damage (63). Third, deficiency of TNF-αR, the specific receptor of TNF-α that is essential for mediating its biological function; injection of TNF-α antibody; or knockout of the TNF-α gene significantly reduces the severity of mouse fallopian tube lesions due to chlamydial infection, illustrating that TNF-α play an important role in immune injury induced by *Chlamydia* (24, 25). Therefore, although TNF-α is not necessary for *Chlamydia* clearance, it can mediate immunopathological damage caused by *Chlamydia*.

## IL

IL, which are produced by lymphocytes, macrophages, and monocytes, and direct certain immune cells to divide and differentiate, consists of several pro- or anti-inflammatory proteins, with pro-inflammatory IL-1β, IL-17A, IL-18 being its best characterized members. The main pro-inflammatory ILs include the IL-1 family, IL-17A, IL-8 (64). While, Major anti-inflammatory cytokines include IL-1 receptor antagonist, IL-4, IL-6, IL-10, IL-11, and IL-13 (65).

### IL-1 Family

The IL-1 family consists of 12 members, including IL-1, IL-18, and IL-33 (66). These ILs are expressed and secreted by different cells, such as mast cells, eosinophils, macrophages, and may play an important role in biological activities such as regulation of immune responses; induction of the inflammatory response, cell proliferation, and differentiation; and promoting the secretion of other cytokines (67). The IL-1 family members are also the central mediators of innate immunity and inflammation (68). These multi-effect cytokines exert a variety of local or systemic responses to viral and bacterial infections and are involved in the pathogenesis of chronic inflammatory diseases (67, 68). This has also been observed in chlamydial infections (69, 70) (**Table 2**).

**Table 2:** IL-1 family in *Chlamydia* infection.

IL-family	Special receptor(Coreceptor)	Functions in *Chlamydi*a infection	Regulating other cytokines
IL-1α	IL-1R1(IL-1R3)	Promote inflammatory responses to *Chlamydia* (11, 70, 71); Tissue lesion (11, 50, 71–73)	Promote IL-6, IL-8
IL-1β	IL-1R2(IL-1R3), IL-1R1(IL-1R3)	Promote inflammatory responses to *Chlamydia* (70, 71, 74–76); Assist to defense against chlamydial infection (74, 77); Drive foam cells formation and accelerate atherosclerosis (73, 78, 79)	Promote IL-6, IL-8
IL-18	IL-1R5(IL-1R7)	Promote inflammatory responses to *Chlamydia* (34, 70, 80); Promote tubal edema (71, 80) and tissue fibrosis (34, 80)	Promote IFN-γ
IL-33	IL-1R4(IL-1R3)	Promote inflammatory responses to *Chlamydia* (70); unclear	Promote Th2 cytokine, suppress Th1 cytokine
IL-1Ra	IL-1R1(NA)	Suppress inflammatory responses to *Chlamydia* (70); unclear	Promote IL-8
IL-36Ra	IL-1R6(IL-1R3)	Unknown	Unknown
IL-36/β/γ	IL-1R6(IL-1R3)	Unknown	Unknown
IL-37	IL-1R5(IL-1R8)	Unknown	Unknown
IL-38	IL-1R6(IL-1R9)	Unknown	Unknown

NA, not applicable.

The increase in IL-8 and IL-1 levels can be detected in the blood and synovial tissue of patients infected with *C. trachomatis* (9). Further, alveolar macrophages and peripheral blood mononuclear cells (PBMCs) obtained from chronic obstructive pulmonary disease (COPD) patients with *C. pneumoniae* infection produce significantly higher amounts of IL-1β and lower amounts of IL-1R-antagonist (10) than cells from COPD patients without *C. pneumoniae* infection produce. In addition, elevated levels of IL-1, IL-6, and IL-8 can be detected in the female cervix with *C. trachomatis* infection (81). Based on these phenomena, IL-1 family might have vital functions in the immune response and acute or chronic inflammation following chlamydial infection (71, 82). Current evidence shows that IL-1α can affect the secretion and maturation of IL-8 during chlamydial infection in two ways. On the one hand, IL-1α combined with DNA of host cells enhances the expression of IL-8, mediating host inflammation in the early stage of chlamydial infection. On the other hand, IL-1α can be released from ruptured cells to bind to IL-1R, promoting the secretion of IL-8 in the later stage of infection (11). Through IL-8 activity, IL-1α might play a role in inflammation and tissue lesions induced by *Chlamydia* (83, 84). IL-1β evolved to assist host defense against chlamydial infection by inducing a wide spectrum of inflammatory cytokines and chemokines, such as IL-6 and IL-8 (71, 74). However, it was found that high expression of IL-1β could drive foam cell formation and accelerate atherosclerosis during *C. pneumoniae* infection (78). Furthermore, IL-1β is believed to be associated with exacerbation of upper genital tract pathology during infection with *C. muridarum* (75). IL-33 can activate ST2-mediated signaling pathways, which mainly promotes Th2 cytokine production (85). Because Th2 cytokines can suppress Th1 responses and inhibit IFN-γ production, it is not likely that IL-33 functions in blocking chlamydial infection (86). It is reported that IL-18 is the only member of the IL-1 family that can induce T, B and NK cells to produce IFN-γ, which is responsible to controlling chlamydial infection (80). However, the clearance of *Chlamydia* did not have significant difference on mouse pneumonitis (MoPn)-infected IL-18 KO mice and wild-type mice (12). Thus, it is speculated that IL-18 may not be necessary for the clearance of chlamydial infection.

The roles of IL-1 members are varied and complex, and their activities and interactions will change as the chlamydial infection progresses. Pathogenic molecules produced by *Chlamydia*, such as pORF5 and Hsp60, can induce the activation of caspase-1 and stimulate secretion of IL-1β, IL-18, and IL-33 through TLR2/myeloid differentiation primary response 88 (MyD88) or NLRP3/ASC/caspase-1 signaling pathways to promote local inflammatory responses to *Chlamydia* (74, 87, 88). Additionally, these proteins may also be related to the pathological mechanism of tubal edema and tissue fibrosis caused by *Chlamydia* (71, 82). It is important to define the role of IL-1β in this context: while this IL protects the host in the defense against chlamydial infection (77, 81), we still believe that it is a promoting disease-related factor in *Chlamydia* infection. IL-18 and IL-33 participate in immunologic injury induced by chlamydial infection, although the precise mechanisms in removing chlamydial are still unknown (11, 72, 89). In short, the IL-1 family not only includes members that regulate inflammation but also includes those are involved in the immunopathological damage caused by *Chlamydia* (72, 77, 87). Doubts still persist regarding the function of some IL-1 cytokines such as IL-37 and IL-38, and further studies are needed to identify their precise roles in chlamydiosis.

### IL-6

Like IL-1, IL-6 has a wide variety of activities related to immune cell functions. IL-6 promotes the terminal differentiation of B cells (90) and T cell survival (91) and helps T cells to overcome suppression by Tregs (92); its most noticeable role is in the defense against infection (90, 93). IL-6 is overexpressed in mice with *Chlamydia*-related tubal factor infertility or humans who suffered from *Chlamydia*-related disease (94, 95). IL-6 can also be detected in the semen and serum of asymptomatic patients with *Chlamydia* infection (96, 97). Elevated IL-6 levels were found to play a protective role in controlling *Chlamydia* infection, given that IL-6 deficient mice were significantly more susceptible to *Chlamydia* infection through the airway than wild-type mice (98, 99). Patients who received anti-IL-6 treatment were also found to be at increased risk of *Chlamydia* infection (13, 100). These results suggest that the role of IL-6 should not be ignored in the inhibition of and therapy for *Chlamydia* infection. There are a few potential explanations for the resistance of IL-6 to *Chlamydia*. IL-8, IL-1β, and other cytokines act as initiators of IL-6 production (101). The MAPK/extracellular regulated protein kinases (ERK) pathway or the Janus kinase (JAK)/signal transducer and STAT pathway mediate the expression and secretion of IL-6 at certain mucosal surfaces or in cell culture supernatants (94, 102). From the perspective of the innate immune response, IL-6 induces the recruitment of white blood cells and promotes apoptosis of neutrophils, mediating inflammation to control infection (93, 103). From the perspective of the adaptive immune response, high expression of IL-6 activates the Th1-like response to clear pathogens by regulating the production of IFN-γ and thus decrease susceptibility to *Chlamydia* infection (94). In addition, TNF-α, which is relevant to the degree of *Chlamydia*-induced fallopian tube obstruction and maintenance of the continuous state of disease, is inhibited by IL-6 in a dose-dependent manner (62, 104). Although IL-6 plays critical roles in controlling *Chlamydia* infections, the effects may vary. Some of the most interesting discoveries are relevant to the varying role of IL-6 in *Chlamydia* infection at different inoculating doses. IL-6 is required for controlling chlamydial infection by limiting replication and colonization by the pathogen, at either high dose or low dose of *Chlamydia muridarum* (*C. muridarum*) (94). In contrast, IL-6 is not essential for induced hydrosalpinx at a high dose of *C. muridarum*, but is required for exacerbating infection-induced hydrosalpinx with low dose of *C. muridarum* (94). The reason might be that IL-6 increases inflammatory infiltration in certain tissues and the specific CD4^+^ and CD8^+^ T cells that produce TNF-α under low-dose chlamydial inoculation (101, 104). These findings also show that host inflammatory responses to IL-6 do not match the extent of the infection.

Besides its role in host defense, IL-6 also mediates the inflammatory pathology. Regardless of infectious dose, the phenomenon correlating with IL-6-induced pathology has been reported mainly in *C. pneumonia* infections and *C. muridarum* mice models (94, 102). Patients with *C. pneumonia*-related COPD were shown to have high levels of IL-6, which exacerbates the disease state and has been proposed to be useful in evaluating the severity of COPD (62, 105). Anti-IL-6 therapy has been used to treat arthritis patients with *C. trachomatis* and *C. pneumonia* infection (14, 100). The possible reason why use anti-IL-6 treatment might be IL-6 participation in the mechanism of chronic inflammatory pathology or the resulting fibrosis (102, 106, 107).

The role of IL-6 in chlamydial infection and the pathological effects thus induced has been controversial. IL-6 mediates inflammation, in terms of both pro-inflammatory and anti-inflammatory functions, in bacterial infection, which is consistent with previous reports (81, 108). Furthermore, while IL-6 promotes host defense against chlamydial infection by balancing inflammatory and immune responses, this IL also exacerbates chlamydial pathogenicity through its involvement in inflammatory pathology. However, the mechanism involved has not yet been fully elucidated and further research is needed (94, 109, 110).

### IL-8

IL-8, a pro-inflammatory chemokine, participates in host defense by recruiting and regulating the activity of immune cells such as leukocytes, basophils, and T lymphocytes (111). IL-8 was found to be increased in the serum of patients suffering from *Chlamydia*-induced pneumonia (112), and has been found to be elevated in the culture supernatant of cervical and colon epithelial cells infected with *Chlamydia* (113). Thus, IL-8 might be related to acute or chronic inflammation after chlamydial infection. Further studies have provided a comprehensive understanding of the function and mechanism of IL-8 in chlamydial infection. Firstly, *Chlamydia* and its pathogenic substances can induce IL-8 secretion by activating the nuclear factor-κB (NF-κB) and MAPK/ERK signaling pathways (114, 115), by activating the IL-10-mediated JAK/STAT signaling pathway (116), or through an IL-1α-mediated IL-1RI-independent mechanism (11). Second, the local hypoxic environment, formed in the process of *C. pneumoniae* infection, also facilitates the secretion of IL-8 in another way (15). These processes are particularly important for continuing the induction of IL-8 at later phases of infection (84). Furthermore, IL-8 is also necessary for chlamydial replication and its synthesis of its components. The trend in up-regulation of IL-8 is consistent with the unique development cycle of *Chlamydia* (113). It is possible that IL-8 promotes the lipid metabolism of host cells, thus enabling the provision of nutrients for chlamydial growth and development (117). *C. pneumoniae* relies on the activation of the IL-8-mediated phosphatidylinositol 3-kinase (PI3K)/protein kinase B (Akt) signaling pathway to stabilize myeloid cell leukemia-1 (MCL-1) and inhibit the spontaneous apoptosis of neutrophils, which act as a transport vehicle and are beneficial for *C. pneumoniae* to establish a productive infection during the initial phase of infection (118). In addition, the IL-8 produced during chlamydial infection in turn reduces the sensitivity of *Chlamydia* to azithromycin and other therapeutic drugs (15). Thus, chlamydial infection and IL-8 form a positive feedback cycle. However, although it is inefficient in resolving chlamydial infection, IL-8 plays a certain role in preventing chlamydial invasion (119). Further research is needed to elucidate the contradictory roles of IL-8 in chlamydial infection. Studies under different infectious conditions may prove valuable.

### IL-17

IL-17, a hallmark cytokine of Th17 cells, performs a pro-inflammatory function and exerts a host-defensive role in many infectious diseases (120, 121). IL-17 is present in inflammatory tissues or the internal environment of almost all patients infected with *Chlamydia* (122, 123). Furthermore, the replicative ability of *Chlamydia* is enhanced in IL-17 KO mice or mice treated with IL-17 inhibitors (17, 124). Thus, IL-17 plays an anti-infective role against *Chlamydia* (125–127). This function is achieved not only by up-regulating inducible nitric oxide synthase (iNOS) production and the cooperative interaction between nitric oxide (NO) and IFN-γ (128), but also *via* the induction of type 1 T cell immunity by DCs (129). Furthermore, the IL-17 response in the early stage was found to be central to amplifying inflammation and initiating host defense against *Chlamydia* through synergy with other cytokines such as IL-6 and macrophage inflammatory protein-2 (MIP-2) (98).

Although IL-17 elicits protection against chlamydial infections *via* its pro-inflammatory function, it can promote inflammatory pathology and participate in the pathogenesis of chlamydial diseases. IL-17 increases local neutrophil infiltration by regulating the expression of chemokines and adhesion molecules in early stage of *Chlamydia* infection (130, 131). It can also drive the secretion of a series of cytokines that cause excessive tissue damage and fibrosis repair (132). The C-Fos/IL-17C signal pathway mediates vascular smooth muscle cell (VSMC) migration and accelerates atherosclerosis resulted from *C. pneumonia* infection (133). Taken together, IL-17 exerts anti-infective effects but is inadequate to clear *Chlamydia* infection. More importantly, it unarguably contributes to the inflammatory pathology of *Chlamydia* infection (134).

### IL-4

IL-4 is crucial for the function of T and B lymphocytes. It can elicit many responses, in particular the humoral immune response that is associated with antibody production (135, 136). IL-4 can be detected in the culture supernatant of PBMCs isolated from patients with *Chlamydia* infection (137, 138). Therefore, IL-4 is considered to play a role in *Chlamydia* infections. It enhances the antigen-presenting ability of B cells by boosting the expression of MHC II, FcϵRII/CD23, and CD40 molecules, thus magnifying immune responses beneficial for eliminating *Chlamydia* (139). This IL-4-mediated enhancement of immune responses also triggers delayed type hypersensitivity (DTH) during *Chlamydia* infection, which might be associated with asthma due to *Chlamydia* (140). Furthermore, IL-4 reduces the secretion of inflammatory cytokines from mononuclear macrophages, inhibiting local tissue damage resulting from excessive Th1 immune responses (141, 142). This explains the previous finding that IL-4 can effectively prevent endometrial injury caused by *C. trachomatis* (18). Based on these data, strongly expressed IL-4 could inhibit *Chlamydia* infection to a certain extent, and also effectively prevent tissue damage. However, the role of IL-4 in *Chlamydia* infection has not been adequately investigated and further elucidation is necessary.

### IL-13

IL-13 has an amino acid homology of 20%-25% with IL-4, thus sharing some, but not all functional properties (143). IL-13 can enhance the host’s resistance to intracellular parasites by enhancing Th2-type cell response (144). However, this appears to differ for *Chlamydia*. The elevated IL-13 levels observed in vaginal secretions and serum after *Chlamydia* infection have been frequently linked to the function of promoting infection and aggravating lesions (145). *In vivo* and *in vitro* experiments indicate, to a certain extent, that IL-13 may enhance *Chlamydia* replication in cell or animal models of infection (16). Together with TNF-α, IL-13 induces certain cells to produce cytokines related to scar formation, such as transforming growth factor-β (TGF-β) (146). In comparison with wild-type mice, *C. muridarum*-infected IL-13 KO mice have faster pathogen clearance, and milder tissue lesions (145). Therefore, it is hypothesized that IL-13 mediates *Chlamydia*-related immunopathology and reflects disease severity after *Chlamydia* infection (16). IL-13 thus has the potential to develop as an evaluation index of *Chlamydia*-induced disease severity. Interestingly, in a mouse model of *C. trachomatis* genital tract infection, two studies have found that a subset of CD4 or CD8 cell populations can produce both IFN-γ and IL-13 (termed CD4γ13 or CD8γ13 T cells, respectively); adoptive transfer of either *Chlamydia*-specific CD4γ13 or CD8γ13 T cells protects oviducts from immunopathology, suggesting a protective role of IL-13 during *Chlamydia* infection (146, 147). The reason for the contradictory result of IL-13 remains unclear. Perhaps IL-13 secreted by innate immune cells in the early stage of infection is mainly associated with pathogenicity, while in the late stage of infection, IL-13 produced by specific T cells, especially those have immune memory, exert protective effect in anti-infection immunity. IL-13 from specific CD4γ13 or CD8γ13 T cells probably is a very small proportion of the total IL-13 produced, so protective IL-13 doesn’t work well in the context of pathogenic IL-13. Knockout of IL-13 gene, but not adoptive transfer of CD4γ13 or CD8γ13 T cells, may affect important immune cells in anti-infection immunity. Anyway, targeted blocking of IL-13 action in *Chlamydia* infection through clarification of cell specific IL-13 mechanisms is a critical goal to aim for in related research.

### IL-10

IL-10 is synthesized by a wide range of cell types such as macrophages, monocytes, Th2, and Treg cells (148). As a potent anti‐inflammatory cytokine, IL-10 not only limits and terminates inflammatory responses, but also plays a crucial role in the control of diseases caused by infectious pathogens; its levels are inversely correlated with disease incidence and severity (149).

The functions of IL-10 in chlamydial infection are however more complex. *Chlamydial* factors such as heat shock proteins 60 (Hsp60) and LPS interact with TLRs to induce high expression of IL-10 from host cells (83, 150). IL-10 can be also secreted from PBMCs infected with *C. trachomatis*, which is dependent on the ERK and p38 signaling pathways (116). It has also been found that the inhibition of TLR2, MyD88, and NF-κB in *C. psittaci-*infected HD11 macrophages significantly reduces IL-10 cytokine production (151). Thus, elevated IL-10 levels can be detected not only in serum, scar tissue homogenates, bronchoalveolar lavage fluid, and cervical secretions from *Chlamydia-*infected hosts (152–154), but also in the supernatant of *Chlamydia*-infected HeLa cells, DCs, and PBMCs (137, 155, 156).

In addition to findings about changes in expression and the possible roles of IL-10 in chlamydial infection, some deeper insights into the roles of IL-10 were gained. Yang and his co-workers revealed that C57BL/6 mice with lower levels of IL-10 had faster clearance of the organism from the lungs after infection with MoPn than BALB/c mice (35). Furthermore, IL-10 KO mice also presented faster clearance of MoPn in lung and genital tract than wild-type mice (157, 158). It’s possible that IL-10 inhibits pro-inflammatory cytokine production for chlamydial clearance by regulating the activation of certain signaling pathways (150, 159). Further, IL-10 down-regulates the expression of MHC I molecules, impairing the presentation of MHC-bacterial epitopes and eliminating activity of CD8^+^ T cells against infected cells, thus reducing the clearance of *Chlamydia* (19). In addition, IL-10 enhances the survival of *Chlamydia* by inhibiting apoptosis of DCs or controlling its antigen presentation function and weakening the immune response (160, 161). Therefore, IL-10 can suppress inflammation-related immune responses against *Chlamydia* species.

Inflammatory processes are responsible for complications induced by *Chlamydia* infections, and IL-10 is hypothesized to be involved in the processes. IL-10 levels in cervical secretions were higher in *C. trachomatis*-infected infertile women than in fertile women (162). The reason could be that IL-10, as an anti-inflammatory factor, reduces local inflammatory infiltration and attenuates the pathological damage such as mice tubal edema due to *C. trachomatis* infection (71). It also decreases the activity of CD8^+^ T cells against infected cells and thereby reduces tissue damage (19). IL-10 is however also responsible for the severe damage, such as tubal infertility and ectopic pregnancy, induced by *Chlamydia* infection (71, 140, 162). These findings are in contrast to the observed mild pathologies induced by IL-10 in *Chlamydia* infection, which may be due to the differences in pathogenesis of *Chlamydia* species in infectious models.

IL-10 is an important factor for balancing the immune system after *Chlamydia* infection, and the modulation of its expression in *Chlamydia*-infected hosts is cautiously performed. Although it can eliminate or promote infection with differing intensity and duration at different infectious phases, the presence of IL-10 decreases *Chlamydia* eradication *via* anti-inflammatory action, and is helpful for controlling and minimizing *Chlamydia*-induced diseases and complications. Moreover, genetic variations in IL-10 gene may be associated with its different expression and the polymorphisms within IL-10 gene may explain interindividual variation in host immune responses to *Chlamydia* infection (150). However, researchers have different opinions on the association between IL-10 polymorphisms and the outcome of *Chlamydia* infections (163). Future investigations regarding the role of IL-10 polymorphisms in *Chlamydia* infections are required.

Members of the IL family play important roles in *Chlamydia* infection, either promoting infection and accelerating the disease, or suppressing *Chlamydia* and alleviating tissue injures. One of our main tasks now is to make the best use of information on ILs for the prevention and treatment of *Chlamydia* infection. A potential method is to identify the function of a certain IL in *Chlamydia* infection by using KO mice, siRNA/chemical inhibition or antibody blockade: this work has already been done for some ILs; second, to generate cells/tissue-specific IL gene-KO mice, and determine the main environmental conditions in which the IL functions optimally. Based on the former two steps, aiming at specific cells/tissues, the relevant IL or antibody/inhibitor of the harmful IL may be used to control *Chlamydia* infection. It might be possible to extend this technique to other diseases in the future.

## Other Cytokines

In addition to the above cytokines, other cytokines such as IL-12, IL-5, and GM-CSF have also been reported in studies on chlamydial infection, although sufficient data are not available. IL-12 coordinates its functions with IFN-γ in *Chlamydia* infection to collectively prevent neonatal pneumonia caused by *C. pneumonia* (164). If either IL-12 or IFN-γ is deficient, the other correspondingly decreases and *Chlamydia* clearance is affected (12, 164). A positive feedback loop exists between the effects of IL-12 and IFN-γ, which strengthens the host’s ability to resist infection. However, excessive IL-12 has a negative impact on the development of CD8^+^ T memory cells, which is not conducive to the host’s resistance to *Chlamydia* reinfection (33). In addition, high levels of IL-5 are closely related to *C. pneumonia*-caused asthma (165). It has also been reported that Th2 type DTH-related immunity and pathology to *Chlamydia* infection is associated with high levels of IL-5 and IL-4 (140). Elevated GM-CSF expression is also observed during *Chlamydia* infection, although the mechanism is not quite clear (166).

## Conclusion

**Figure 2:**
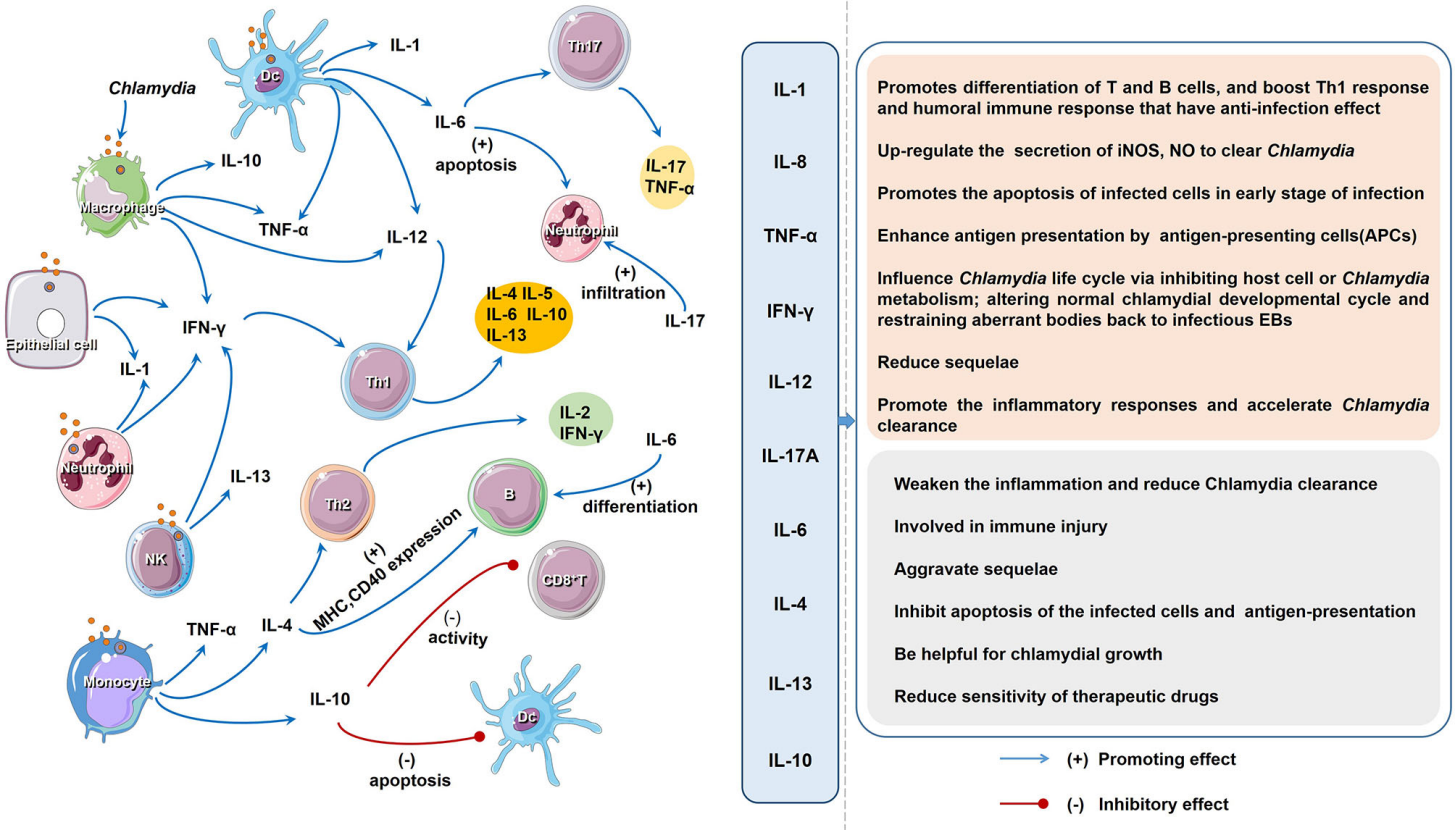
The regulatory network and function of different cytokines upon *Chlamydia* infection. The host cells produce a variety of cytokines after chlamydial infection. Cytokines induce cell immunity response and produce more cytokines. As descripted in text, the impact of all the cytokines on the host chlamydial infection can be divided into two aspects: favorable and unfavorable.

Here, we summarized the effects and interactions of important cytokines involved in Chlamydia infection (**Figure 2**), and offered some valuable insights into the potential mechanisms and proposed countermeasures. A range of factors contribute to cytokine production during Chlamydia infection (**Supplementary Table 1**). First, *Chlamydia* species (with different components), the number of *Chlamydia* (or dose of their products) and the route of infection are implicated in cytokine production (167). A certain chlamydial strain and the corresponding products exert effects on their cytokine production, which related to the varied toxicity in host (168). For example, plasmid might partially explain the different pathological features and cytokine production in plasmid-deficient and plasmid-competent *Chlamydia* (169). In *C. trachomatis* D/UW-3/CX strain, MOMP, CPAF and HSP60 are all important cytokine inducers, while CPAF and MOMP are more potent in triggering IL-1β, as compared to HSP60 (109). Second, major factors influencing cytokine variation in hosts include host genetics, non-heritable factors and the microbiome (167). Host genetic variation accounts for a significant part of variability in cytokine production by different strains of mice. For example, 27 oviduct cytokines were significantly higher in highly susceptible (hydrosalpinx) CBA/J than those of resistant A/J mice (170). 16 oviduct cytokines were significantly higher in C5-competent than those of C5-deficient mice (171). Some cytokine coding genes are known to be highly polymorphic, which are also regarded as a possible cause for difference in the cytokine production and contribute to various pathological conditions (172). In addition, non-heritable factors, including age, body weight and gender, and gut microbiome may also engage in variation in cytokine production during *Chlamydia* infection (167). The variability of cytokine production makes their biological function to be systemic or tissue-specific, complementary (several cytokines work together) or pleiotropic (a cytokine with different functions). Further identifying the causes and consequences of variation in cytokine production is a crucial step to better understand the pathogenesis of *Chlamydia* infection.

In chlamydial infection, the behavior of some cytokines is a double-edged sword in inflammation and immune-mediated pathogenesis (**Figures 1**, **2**; **Table 2**). Although the role of cytokines at different stages of *Chlamydia* infection has been extensively studied, still some important questions warrant further exploration. For instance, how do different cytokines coordinate their roles in chlamydial pathogenesis? Why do cytokines from different cells play different roles in chlamydial infection (32, 173)? The intestinal tract, which is colonized by more virulent *Chlamydia* strains and a higher chlamydial burden, might also take part in the pathogenesis and development of chlamydial diseases. Whether cytokines from innate lymphoid cells (ILCs), especially ILC3 play a role in *Chlamydia* caused diseases. How does gut microbiota affect the host cytokine network during chlamydial infection? How to dynamic remodeling cytokine network for clearing *Chlamydia* infection or promoting body recovery? With the solution of these important questions, we believe that manipulation of key cytokines in chlamydial infection will represent a novel strategy to treat chlamydial diseases.

## Author Contributions

WX conceived of the presented idea and drafted the manuscript. ZZ, NY, XL and ST gave some idea to draft the manuscript. ZZ, AL, and LH critically revised the manuscript. All authors contributed to the article and approved the submitted version.

## Funding

This work was supported by the National Natural Science Foundation of China (No. 31570179), the Hunan Natural Science Foundation (No. 2020JJ4084), the Hunan Provincial Key Laboratory for Prevention and Control of Special Pathogens (No. 2014-5), the Hunan Province Cooperative Innovation Center for Molecular Target New Drug Study (2015–351), and the Hunan Provincial Key Discipline Project (No. 2011-76).

## Conflict of Interest

LH was employed by Huang Nanyue Biopharmaceutical Co. Ltd.

The remaining authors declare that the research was conducted in the absence of any commercial or financial relationships that could be construed as a potential conflict of interest.
