# Supplementary material for: Insights Into Host Cell Cytokines in Chlamydia Infection
Source: Front Immunol. 2021 May 21;12:639834. doi: 10.3389/fimmu.2021.639834 (PMC8176227; doi:10.3389/fimmu.2021.639834)
Supplement: Supplementary file 1 [file Table_1.docx]

Supplementary tables1 Variation in cytokine production during *Chlamydia* infection

| **Strain of *Chlamydia*** | | **Infected host/cell** | **The route and dose of infection** | **Effect on cytokine production** | **Significance and perspectives** | **Reference** |
| --- | --- | --- | --- | --- | --- | --- |
| ***Chlamydia trachomatis***  **Ocular tropic strain** | **A/HAR-13** | HeLa | in vitro infection,  MOI= 1 | CCL5(48h), IL-1α(48h), IL-18(24h), CXCL1(48h)/ MIF(24, 48h) | The host cytokine response to *C. trachomatis* is cell type and serovar specific. | (1) |
|  |  | HVEC |  | CCL5(48h), IL-6(48h), GM-CSF(48h), CXCL1(48h)/ IL-1ra (48h), MIF(48h） |  |  |
|  |  | THP1 |  | IL-1β(48h), IL-8(24,48h), MIP-1α/β(24,48), CXCL1(24h), CXCL10(24,48h)/ IL-6(24h), IL-16(24h), MIF(24h) |  |  |
|  |  | HCjE |  | C-CSF(24h), CXCL1(24h), CXCL10(48h)/ IL-8(24h), MIF (48h) |  |  |
|  |  | HFK-2 |  | IL-1β(48h), IL-8(48h), CXCL1(48h)/ IL-1β(24h) |  |  |
|  |  | HCK­ |  | IL-1α(48h), IL-1β(48h), IL-6(48h), GM-CSF (48h), CXCL1 (48h), IL-ra(48h) |  |  |
|  |  | Human being | eye-hand-eye infection | TNF-α,IFN-γ, IL-1α, IL-1β，IL-8 | Cytokine lead to pathological inflammatory response, like conjunctival fibrosis and scarring. | (2) |
| **Chlamydia trachomatis**  **Genitourinary tract trop strain** | **D/UW-3/CX** | HeLa | in vitro infection,  MOI= 1 | IL-1α(24h), IL-8(48h), IL-18(48h)/ IL-8(12h), MIF(48h) | The host cytokine response to *C. trachomatis* is cell type and serovar specific.  Ectocervical keratinocytes and non-keratinized vaginal epithelial cells may play an integral role in initiating the early innate response to *Chlamydia* infection and provide a major barrier to infection of more permissive tissues. | (1) |
|  |  | HVEC |  | IL-6(48h), GM-CSF(48h) |  |  |
|  |  | THP1 |  | IL-1α(24h), IL-1β (48h), IL-8(24h), CXCL1/10(48h) / IL-6 (24h), IL-16(24h) |  |  |
|  |  | HCjE |  | G-CSF(24h), CXCL1(24h), CXCL10(48h)/ IL-8(24h), MIF (48h） |  |  |
|  |  | HFK-2 |  | IL-1β(48h), IL-8(48h), CXCL1(48h) / IL-1β(24h), ICAM-1 (24h) |  |  |
|  |  | HCK |  | GM-CSF(24h), IL-6(24h), IL-1ra(48h) |  |  |
|  |  | Human being | sexual transmission | IL-1α, IL-1β，IL-8, GM-CSF,TNF-α,IFN-γ | Contribute to tubal edema, fibrosis | (3) |
|  | **MOMP**  **(D/UW-3/CX)** | P- vs P+ | in vitro stimulation | TNF-α(HSP60> CPAF >MOMP),  IL-1β(MOMP>CPAF>HSP60),  IL-6(no obvious difference)­,  TNF-α, IL-1β, IL-6(plasmid- bearing *Chlamydia*-infected > plasmid-free *Chlamydia*) | MOMP, HSP60, CPAF, as important chlamydial pathogenic substances, are potent in triggering proinflammatory cytokines production. | (4) |
|  | **CPAF**  **(D/UW-3/CX)** |  |  |  |  |  |
|  | **HSP60**  **(D/UW-3/CX)** |  |  |  |  |  |
| ***Chlamydia trachomatis***  **Lymphogranuloma venereum strain**  **(LGV)** | **L2/434-B** | HeLa | in vitro infection,  MOI= 1 | IL-18(24,48h), IL-1α(24,48h), IL-6(48h), CXCL1/10(48h) | ①The host cytokine response to *C. trachomatis* is cell type and serovar specific; ②The induced cytokines by LGV infection might be related with chlamydial ability to survive in macrophages and cause systemic disease; ③L2 trigger chemotactic factors to enhance macrophage recruitment to the site of infection. | (1) |
|  |  | HVEC |  | GM-CSF(24h), IL-6(48h)/ CXCL1/10(48h), IL-18(48h), MIF(48h), |  |  |
|  |  | THP1 |  | CXCL10(24,48h), IL-1β (48h), IL-1α(24h), IL-8(24h), CXCL1(24,48h) / IL-6 (24h), IL-16(48h), IL-18(48h), IL-8(24,48h), |  |  |
|  |  | HCK |  | G-CSF(48h), CXCL1(48h), IL-6(48h) |  |  |
|  |  | HCjE |  | IL-1β(48h), IL-8(48h), CXCL10(48h)/ IL-1β(24h) |  |  |
|  |  | HFK-2 |  | GM-CSF(24,48h), G-CSF(24,48h), IL-6(24,48h), IL-1β (48h), IL-8(24,48h), CXCL1(24h)/ CXCL10(48h) |  |  |
|  |  | Human being | sexual transmission | IL-1α, IL-1β，IL-8, GM-CSF,TNF-α,IFN-γ/ IL-6 | Involved in pathogenesis of LGV, inducing inflammation-related buboes, fibrosis, damage of urogenital mucosal, and scarring | (5) |
| ***Chlamydia pneumoniae*** | **TW-183** | A549/HEp-2 | in vitro infection, MOI=1  centrifuge vs non centrifuge | IL-1β, IFN-γ, TNF-α.  centrifugation can up-regulate IL-1β | The centrifugation condition for infection also has an effect on the production of cytokines | (6, 7) |
|  |  | Human or mice macrophages | in vitro infection,  MOI=3 | IL-1β, IFN-γ, TNF-α, IL-8 | The cytokine might enhance macrophage recruitment to the site of infection, promoting Th1 response. | (8) |
|  |  | Human being | transmitted by droplets and respiratory secretions | IFN-γ, TNF-α, IL-1β, IL-1α | Contribute to inflammations-related①respiratory diseases, e.g. atypical pneumonia, bronchitis, pharyngitis, asthma; ②non-respiratory diseases, e.g. vasculitis, encephalitis. | (9) |
|  |  | C57BL/6J mice | I.n,3×10^7^IFU/mouse | IFN-γ, TNF-α, IL-1β, IL-1α | Induce atypical pneumonia. | (10) |
|  | **MOMP (TW-183)** | HEp-2 | in vitro stimulation | IL-1α, IL-1β, TNF-α | MOMP, HSP60 are important inducer for proinflammation cytokines. | (11) |
|  | **HSP60 (TW-183)** | HEp-2 | in vitro stimulation | TNF-α, IL-1β, IL-8 |  | (12) |
| ***Chlamydia muridarum*** | **Nigg strain** | CBA/J vs A/J mice | I.v, 2×10^5^ IFU/mouse | 27 oviduct cytokines including IL-6, IL-17, G-CSF were significantly higher in CBA/J than those of A/J mice | The host cytokine response to *Chlamydia* is related to host genetic background. | (13) |
|  | **Nigg strain** | C5-deficient vs  C5-competent  mice | I.u, 2×10^5^ IFU/mouse | 16 oviduct cytokines including IL-1β, IL-1α, IL-2, and KC were significantly higher in C5-competent mice than those of C5-deficient mice. | C5 contribute to chlamydial  induction of hydrosalpinx by  enhancing inflammatory response | (14) |
|  | **G28 vs G0** | C3H/HeJ mice | I.v, 2×10^5^ IFU/mouse | 12 oviduct cytokines including IL-1β, IL-1α and IL-12 were significantly higher in mice infected with CMG0 than in those infected with CMG28. | The attenuated strain CMG28 might contain genes that regulate cytokine production and inflammation during *C. muridarum* infection. | (15) |
|  | **Plasmid-free** | CBA/J mice | I.u vs, I.v,  2×10^5^ IFU/mouse | 12 oviduct cytokines including IL-1α, IL-12 and MIG were significantly higher in mice after intrauterine infection with *C. muridarum* than those from intravaginal infection. | The routes of infection can also influence cytokine production during chlamydial infection. | (16) |
| **Chlamydia**  **psittaci** | **DC15** | C3aR-deficient vs  C3aR-competent  mice | I.n, 4×10^4^ IFU/mouse | IFN-γ, TNF, MCP-1, IL-6, and IL-10 were elevated in lung homogenate and BLAF. C3aR-competent mice get back to norm the second week after infection, while C3aR−/−mice showed prolonged elevated cytokine levels. | C3aR is a host factor for  modulating cytokine production in | (17) |
|  | **CPSIT_P7**  **(6BC plasmid**  **encoded)** | HEp-2 | in vitro stimulation | GM-CSF, IL-6, L-8, and MCP-1 up-regulate, in a concentration- and time-dependent manner. | *C. psittaci* plasmid-encoded CPSIT_P7 can elicit cytokine production | (18) |

HVEC: human vaginal epithelial cells; HCK: human ectocervical keratinocytes; HCjE: Immortalized human conjunctival epithelial cells; HFK-2: human foreskin keratinocytes; BALF: Broncho-alveolar Lavage Fluid; P-: PBMCs from plasmid-free chlamydia-infected patients; P+: PBMCs from plasmid-bearing chlamydial-infected patients. The cytokine in red letter means elevated cytokine, the cytokine in blue letter means reduced cytokine.

1. Faris R, Andersen SE, McCullough A, Gourronc F, Klingelhutz AJ, Weber MM. Chlamydia trachomatis Serovars Drive Differential Production of Proinflammatory Cytokines and Chemokines Depending on the Type of Cell Infected. *Front Cell Infect Microbiol* (2019) 9:399. Epub 2020/02/11. doi: 10.3389/fcimb.2019.00399. PubMed PMID: 32039039; PubMed Central PMCID: PMCPMC6988789.

2. Hu VH, Holland MJ, Burton MJ. Trachoma: protective and pathogenic ocular immune responses to Chlamydia trachomatis. *PLoS Negl Trop Dis* (2013) 7(2):e2020. Epub 2013/03/05. doi: 10.1371/journal.pntd.0002020. PubMed PMID: 23457650; PubMed Central PMCID: PMCPMC3573101.

3. Elwell C, Mirrashidi K, Engel J. Chlamydia cell biology and pathogenesis. *Nat Rev Microbiol* (2016) 14(6):385-400. doi: 10.1038/nrmicro.2016.30. PubMed PMID: WOS:000376164300011.

4. Cheong HC, Lee CYQ, Cheok YY, Shankar EM, Sabet NS, Tan GMY, et al. CPAF, HSP60 and MOMP antigens elicit pro-inflammatory cytokines production in the peripheral blood mononuclear cells from genital Chlamydia trachomatis-infected patients. *Immunobiology* (2019) 224(1):34-41. Epub 2018/11/28. doi: 10.1016/j.imbio.2018.10.010. PubMed PMID: 30477893.

5. Jendro MC, Raum E, Schnarr S, Kohler L, Zeidler H, Kuipers JG, et al. Cytokine profile in serum and synovial fluid of arthritis patients with Chlamydia trachomatis infection. *Rheumatol Int* (2005) 25(1):37-41. Epub 2003/11/01. doi: 10.1007/s00296-003-0393-1. PubMed PMID: 14593490.

6. Yang J, Hooper WC, Phillips DJ, Tondella ML, Talkington DF. Induction of proinflammatory cytokines in human lung epithelial cells during Chlamydia pneumoniae infection. *Infect Immun* (2003) 71(2):614-20. Epub 2003/01/24. doi: 10.1128/iai.71.2.614-620.2003. PubMed PMID: 12540537; PubMed Central PMCID: PMCPMC145357.

7. Quinn TC, Gaydos CA. In vitro infection and pathogenesis of Chlamydia pneumoniae in endovascular cells. *Am Heart J* (1999) 138(5 Pt 2):S507-11. Epub 1999/10/28. doi: 10.1016/s0002-8703(99)70287-5. PubMed PMID: 10539860.

8. Takaoka N, Campbell LA, Lee A, Rosenfeld ME, Kuo CC. Chlamydia pneumoniae infection increases adherence of mouse macrophages to mouse endothelial cells in vitro and to aortas ex vivo. *Infect Immun* (2008) 76(2):510-4. Epub 2007/12/12. doi: 10.1128/IAI.01267-07. PubMed PMID: 18070891; PubMed Central PMCID: PMCPMC2223438.

9. Boelen E, Steinbusch HWM, Bruggeman CA, Stassen FRM. The Inflammatory Aspects of Chlamydia Pneumoniae-Induced Brain Infection. *Drug Today* (2009) 45:159-63. PubMed PMID: WOS:000272015100023.

10. Sommer K, Njau F, Wittkop U, Thalmann J, Bartling G, Wagner A, et al. Identification of high- and low-virulent strains of Chlamydia pneumoniae by their characterization in a mouse pneumonia model. *FEMS Immunol Med Microbiol* (2009) 55(2):206-14. Epub 2008/12/17. doi: 10.1111/j.1574-695X.2008.00503.x. PubMed PMID: 19076226.

11. Confer AW, Ayalew S. The OmpA family of proteins: roles in bacterial pathogenesis and immunity. *Vet Microbiol* (2013) 163(3-4):207-22. Epub 2012/09/19. doi: 10.1016/j.vetmic.2012.08.019. PubMed PMID: 22986056.

12. Costa CP, Kirschning CJ, Busch D, Durr S, Jennen L, Heinzmann U, et al. Role of chlamydial heat shock protein 60 in the stimulation of innate immune cells by Chlamydia pneumoniae. *Eur J Immunol* (2002) 32(9):2460-70. Epub 2002/09/11. doi: 10.1002/1521-4141(200209)32:9<2460::AID-IMMU2460>3.0.CO;2-M. PubMed PMID: 12207330.

13. Chen J, Zhang H, Zhou Z, Yang Z, Ding Y, Zhou Z, et al. Chlamydial induction of hydrosalpinx in 11 strains of mice reveals multiple host mechanisms for preventing upper genital tract pathology. *PLoS One* (2014) 9(4):e95076. Epub 2014/04/17. doi: 10.1371/journal.pone.0095076. PubMed PMID: 24736397; PubMed Central PMCID: PMCPMC3988139.

14. Yang Z, Conrad T, Zhou Z, Chen J, Dutow P, Klos A, et al. Complement factor C5 but not C3 contributes significantly to hydrosalpinx development in mice infected with Chlamydia muridarum. *Infect Immun* (2014) 82(8):3154-63. Epub 2014/05/21. doi: 10.1128/IAI.01833-14. PubMed PMID: 24842924; PubMed Central PMCID: PMCPMC4136226.

15. Chen C, Zhou Z, Conrad T, Yang Z, Dai J, Li Z, et al. In vitro passage selects for Chlamydia muridarum with enhanced infectivity in cultured cells but attenuated pathogenicity in mouse upper genital tract. *Infect Immun* (2015) 83(5):1881-92. Epub 2015/02/26. doi: 10.1128/IAI.03158-14. PubMed PMID: 25712926; PubMed Central PMCID: PMCPMC4399068.

16. Chen J, Yang Z, Sun X, Tang L, Ding Y, Xue M, et al. Intrauterine infection with plasmid-free Chlamydia muridarum reveals a critical role of the plasmid in chlamydial ascension and establishes a model for evaluating plasmid-independent pathogenicity. *Infect Immun* (2015) 83(6):2583-92. Epub 2015/04/15. doi: 10.1128/IAI.00353-15. PubMed PMID: 25870225; PubMed Central PMCID: PMCPMC4432736.

17. Dutow P, Fehlhaber B, Bode J, Laudeley R, Rheinheimer C, Glage S, et al. The complement C3a receptor is critical in defense against Chlamydia psittaci in mouse lung infection and required for antibody and optimal T cell response. *J Infect Dis* (2014) 209(8):1269-78. Epub 2013/11/26. doi: 10.1093/infdis/jit640. PubMed PMID: 24273177; PubMed Central PMCID: PMCPMC3969542.

18. Chen Q, Li Y, Yan X, Sun Z, Wang C, Liu S, et al. Chlamydia psittaci Plasmid-Encoded CPSIT_P7 Elicits Inflammatory Response in Human Monocytes via TLR4/Mal/MyD88/NF-kappaB Signaling Pathway. *Front Microbiol* (2020) 11:578009. Epub 2020/12/22. doi: 10.3389/fmicb.2020.578009. PubMed PMID: 33343522; PubMed Central PMCID: PMCPMC7744487.
